# Who Uses Paid Help? Racial/Ethnic Differences in Caregiving Networks

**DOI:** 10.1093/geroni/igaf122.064

**Published:** 2025-12-31

**Authors:** Wen-Hua Lai, Wenjing Li, Natasha Nemmers, Amanda Leggett

**Affiliations:** Wayne State University, Detroit, Michigan, United States; Wayne State University, Detroit, Michigan, United States; Wayne State University, Detroit, Michigan, United States; Wayne State University, Detroit, Michigan, United States

## Abstract

Racial/ethnic disparities in residential care arrangements are well-documented, with Black and Hispanic older adults relying more on unpaid family caregivers and less on long-term care than Whites. However, less is known about racial/ethnic differences in using paid helpers alongside unpaid family caregivers among community-dwelling older adults. Using longitudinal data from the National Health and Aging Trends Study (2011-2019; N = 6,876), we applied survey-weighted mixed-effects logistic regression models to examine racial/ethnic differences in whether paid helpers were utilized. We assessed caregiving network size, care needs, and dementia status as covariates, adjusting for sociodemographic characteristics and chronic health conditions. Hispanic older adults had the highest prevalence of paid helpers (13.8%), slightly above Blacks and Whites (both 11%). Individuals with dementia had increased odds of paid helper use, with Blacks (possible dementia) and Hispanics (probable dementia) more likely than Whites to use paid helpers. Larger caregiving networks generally increased paid helper use, but Blacks and Hispanics with larger networks had 20-24% lower odds than Whites. Specific care needs (self-care, mobility, medication management) increased odds of paid helper use, with Blacks (self-care/mobility needs) and Hispanics (medication, self-care, mobility needs) having higher odds than Whites. Overall, Hispanics and Blacks were more likely to utilize paid help when caregiving networks were smaller or care needs greater, but reliance on paid help decreased as networks expanded. This suggests that family or friend caregivers may compensate for paid support, reflecting cultural preferences for informal care. Findings can inform targeted interventions and policies promoting equitable and culturally sensitive care coordination.

